# The association of medication-related osteonecrosis of the jaw with *Actinomyces spp*. infection

**DOI:** 10.1038/srep31604

**Published:** 2016-08-17

**Authors:** Guenter Russmueller, Rudolf Seemann, Kathrin Weiss, Victoria Stadler, Manuel Speiss, Christos Perisanidis, Thorsten Fuereder, Birgit Willinger, Irene Sulzbacher, Christoph Steininger

**Affiliations:** 1 Department of Oral- and Maxillofacial Surgery, Medical University of Vienna, Austria; 2 Department of Medicine I, Medical University of Vienna, Austria; 3 Division of Clinical Microbiology, Department of Laboratory Medicine, Medical University of Vienna, Austria; 4 Department of Pathology, Medical University of Vienna, Austria

## Abstract

Medication-related osteonecrosis of the jaw (MRONJ) represents a complication of bisphosphonate treatment that responds poorly to standard treatment. In a retrospective cohort study we investigated a possible role of *Actinomyces spp.* in the pathogenesis of MRONJ. Deep biopsies of necrotic bone were collected during surgical treatment of MRONJ and evaluated by histology and microbiology for the presence of *Actinomyces spp*. Microbiological, demographic and clinicpathological data were analyzed for risk of *Actinomyces*-associated MRONJ. Between 2005 and 2014, 111 patients suffering from histologically-confirmed MRONJ were identified. *Actinomyces spp.* were detected in 99 cases (89%) by histology and in six further patients by microbiological culture. A diverse microbial flora was found in all specimens without association with *Actinomyces spp.* Demographic and clinicopathological characteristics did not separate significantly *Actinomyces*-positive from *Actinomyces*-negative cases. Our observations confirm previous reports of a high prevalence of *Actinomyces spp.* in MRONJ in the single largest cohort available up to now. The high prevalence of Actinomyces *spp*. and the lack of clinicopathological risk factors underline the prominent role of *Actinomyces spp*. in MRONJ and may change the current understanding of MRONJ. Established prolonged antimicrobial treatment regimens against *Actinomyces spp.* infection could therefore be a mainstay of future MRONJ management.

Medication-related osteonecrosis of the jaw (MRONJ) is a rare but difficult to treat complication of bisphosphonate treatment. Incidence of MRONJ ranges between 0.1% and 10% in patients with cancer with clearly higher rates observed in investigator-initiated, academic studies^1^. MRONJ is characterized by necrosis of the maxilla and mandible that leads to superficially exposed bone without a tendency of spontaneous healing for more than eight weeks. The diagnosis is established on basis of a history of recent treatment with an antiresorptive drug in absence of a history of radiotherapy to the jaws^2^. In most cases the onset of MRONJ is triggered by a dental procedure affecting the oral mucosa or alveolar bone^3^. Signs and symptoms may occur before the development of clinically detectable MRONJ including tooth mobility, prolonged jaw pain, gingival swelling, erythema, and ulceration^4^. Fistulae may develop upon secondary infection and are associated with significantly increased morbidity and reduced quality of life. Management of MRONJ is based on discontinuation of bisphosphonate administration, antimicrobial treatment and surgical resection of necrotic bone^2^. Resolution of MRONJ, however, may be achieved only in 30% of patients and clearly underlines the urgent need for more effective prophylactic and therapeutic strategies^5^.

The causal link between MRONJ and administration of bisphosphonates was established with the rapidly increasing number of case series and observational studies that substantiated early evidence^1^. Accordingly, warnings about the risk of MRONJ in patients with cancer were issued by health authorities and manufacturers of bisphosphonates. A better understanding of the pathophysiology underlying the evolution of MRONJ, however, would facilitate development of more efficient preventive strategies. Intravenous bisphosphonates are important treatment options for preventing skeletal related events and to prolong survival in a diversity of generalized or disseminated bone diseases including malignancy, osteoporosis, Paget’s disease, and metastatic bone disease of multiple myeloma, breast, lung, and prostate cancer^5^,^6^. On the other hand, prolonged and high-dose treatment with intravenous bisphosphonates as well as combination treatment with anti-angiogenetic drugs were identified as significant risk factors for MRONJ^7^,^8^.

Still, bisphosphonate treatment may not explain exclusively the observed predilection of the jaw of cancer patients^1^. Remodeling or oversuppression of bone resorption, inhibition of blood supply, constant microtrauma, dentoalveolar surgery, or local inflammation were proposed in association with bisphosphonate treatment to explain the unique localization to the jaw but none of these hypotheses explain all cases^9^. Evidence suggesting a role of *Actinomyces species* in the evolution of MRONJ accumulated over the past years, but these small case series and reviews revealing a high prevalence of *Actinomyces spp.* in MRONJ did not had impact on current treatment recommendations up to now^10^,^11^,^12^,^13^.

*Actinomyces spp.* are Gram-positive, facultative anaerobic, non-spore-forming and commonly filamentous microorganisms. *Actinomyces spp.* are commensals of the mucosa of oropharynx, gastrointestinal tract and female genital tract. Nevertheless, in case of breaches to the mucosal barrier by trauma, surgical procedures, or foreign bodies, microbes may invade deep tissue structures and cause a difficult to treat, chronic-progressive disease termed Actinomycosis. Standards of treatment for invasive actinomycosis have been developed, validated and adapted during the past five decades and is based on prolonged antimicrobial treatment for 2–6 months combined with surgery. The anatomic region affected (e.g. cervicofacial, pulmonal, abdominal) is not associated with a requirement to adapt antimicrobial treatment duration^14^,^15^.

The aim of the present study was to evaluate in a large and well-characterized cohort of patients suffering from MRONJ the incidence of *Actinomyces spp.* infection and possible risk-factors that may predispose patients to this infection. Contrary to expectation, a large majority of the present MRONJ cases was associated with *Actinomyces spp.* infection.

## Materials and Methods

### Study population

The present retrospective study of Actinomycosis associated with MRONJ included all consecutive patients suffering from MRONJ and who were treated at the Department of Oral- and Maxillofacial Surgery, at the Medical University of Vienna, between 2005 and 2014. Inclusion criteria were clinically diagnosed MRONJ according the current guidelines of the American Association of Oral and Maxillofacial Surgeons (AAOMS)^2^, current or previous treatment of malignancy or osteoporosis with a bisphosphonate. Accordingly, the clinical diagnosis of MRONJ was defined as presence of exposed necrotic bone of maxilla or mandible, with or without pain and signs and symptoms of superinfection, for more than eight weeks without tendency of spontaneous healing. Patients were excluded from the study when having received radiotherapy, bone biopsy was not available for histological evaluation, or the clinically suspected diagnosis could not verified by histology. Staging of confirmed cases of MRONJ was done in respect of clinical symptoms and severity of the disease from Stage 0 to Stage 3 according to AAOMS guidelines^2^. Stage 0 cases were not included as they are characterized by non-specific symptoms or radiographic findings without featuring exposed necrotic bone. Treatment of the Stage 1 to 3 MRONJ cases followed an established algorithm combining conservative pre-treatment followed by surgical removal of necrotic bone and soft tissue closure. Sample collection for histology was performed in all cases during the surgical standard procedure. According to this standard treatment algorithm, all patients received systemic antibiotic treatment with amoxicillin (2 × 1 g/24 hours)/clavulanic acid (2 × 500 mg/24 hours) or clindamycin (3 × 300 mg/24 hours) for approximately 4 weeks between admittance and surgery^16^,^17^.

Demographic, clinicopathological and follow-up data were extracted retrospectively from the Vienna General Hospital Patient Information System (AKIM). The study protocol was approved by the Ethics Committee of the Medical University of Vienna, Austria (ECS 1824/2015) and all study related procedures were done according to the declaration of Helsinki. Due to the retrospective cohort study design, no informed consent had to be obtained by approval of the ethics committee.

### Histological and microbiological analysis

The specimens retrieved under surgical treatment of MRONJ comprised of deep bone samples for analysis by histology and microbiology in all cases. The respective specimens were divided into two aliquots and either fixed in 4% phosphate-buffered saline (PBS)-formalin and decalcified with use of ethylenediaminetetraacetic acid (EDTA) for 7 days for histological analysis or immediately transferred into reduced transport medium (Port-A-Cul™, Becton, Dickinson and Company, NJ, USA) and transported to the microbiological laboratory for microbiological culture.

**Figure 1:**
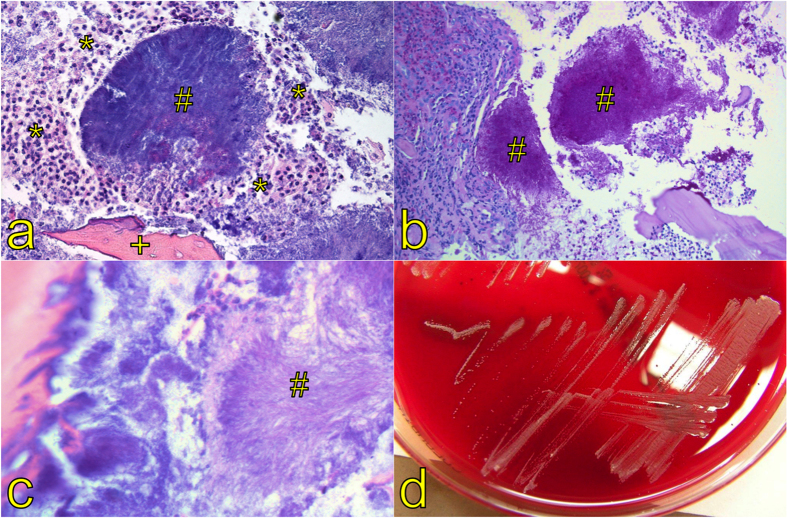
H&E staining of an aggregate composed of *Actinomyces spp.* filaments, so called sulfur granules (#), which macroscopically appear as yellow granules, surrounded by neutrophilic granulocytes (*) and a necrotic bone trabecula (+, magnification x100) (**a**). The granules (#) stain PAS positive (magnification x200) (**b**). High magnification elucidates the filamentous structure (#, sun-ray morphology) of the organisms (magnification x400) (**c**). Typical growth pattern of *Actinomyces spp.* in microbiological culture (**d**).

For histological analysis, specimens were then embedded in paraffin, cut into 2 μm thick slices and stained by hematoxylin and eosin (H&E). To highlight *Actinomyces spp.*, the specimens were stained additionally by periodic acid-Schiff (PAS), Gram and Grocott’s methenamine silver stain (GMS). The slides were examined by a trained pathologist for pathognomonic features of actinomycosis (sulfur granules) and photographed by light microscopy (Fig. 1)^18^,^19^.

For microbiological culture, specimens were streaked to blood agar, chocolate agar, and Sabouraud Dextrose-Agar and were incubated at 29°–31 °C as well as 35°–37 °C. For the growth of anaerobic bacteria, Brucella agar (Becton Dickinson, Heidelberg, Germany) was inoculated and incubated at 35°–37 °C under anaerobic conditions for 21 days in total. In case of growth, identification was performed either biochemically using Vitek^®^ (bioMérieux, Marcy l’Etoile, France) or MALDI-TOF (Bruker, Bremen, Germany). If these methods were unsuccessful, isolates were identified by 16S sequence analysis (Fig. 1).

### Statistical analysis

The primary endpoint of this study was the prevalence of MRONJ-related actinomycosis within the study population as confirmed by histology. Secondary objectives were to determine clinicopathological risk factors for the development of MRONJ-related actinomycosis by means of logistic regression models. The models including one factor were compared to the null model using a likelihood ratio test and thus providing the p-values. The distribution of bisphosphonate drugs in the actinomyces positive and the negative group was tested for count distribution by means of a chi-squared test instead of applying logistic regression. Furthermore, a Fisher’s exact test was used to investigate the allocation of microbiological culture results between actinomyces positive and the negative cases in histology. A p-value of <0.05 was defined as statistically significant. All calculations were performed using the statistical programming environment “R” (version 2.15.1, Vienna, Austria). Finally all p-values were corrected for multiple testing using Bonferroni adjustment.

## Results

### Demographic, clinical and histological results

A total of 150 patients fulfilling the clinical criteria of MRONJ were identified during the study period of 2005 to 2014. Of these patients, 39 cases were excluded from the study because of a previous history of radiotherapy (n = 6), unavailable bone specimen for histological analysis (n = 24), or histological evaluation not supporting the clinical diagnosis (n = 9). The final cohort of 111 patients with clinically diagnosed and histologically confirmed MRONJ were predominantly female, above the age of 65 years, and all were Caucasian (Table 1). The majority of MRONJ patients had a malignancy as underlying disease and most of them suffered from malignant bone disease. MRONJ was diagnosed in most patients at a clinical stage 2 (n = 55) or stage 1 (n = 43). Thirteen patients had stage 3 disease. The predominant location of MRONJ was in the mandibular bone (n = 72), followed by the maxilla (n = 34) or both (n = 5).

**Figure 2:**
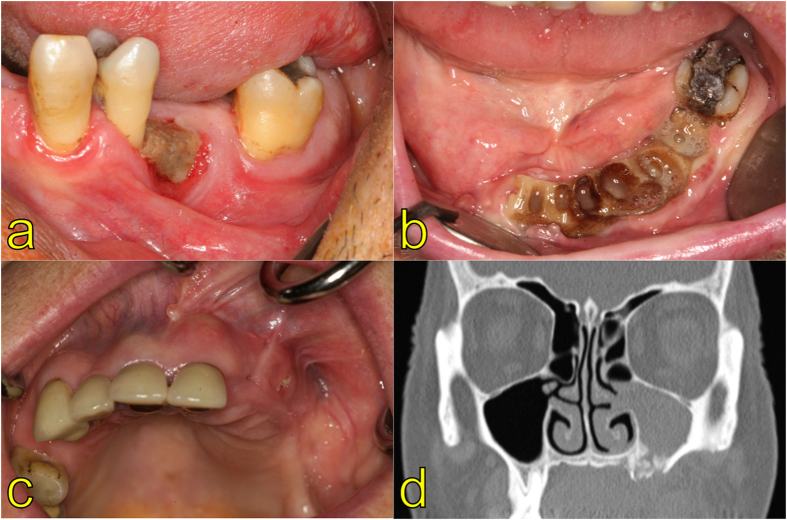
Clinical findings in patients suffering from MRONJ showing necrotic alveolar bone (Stage 1) distal to a premolar tooth (**a**) or large areas of exposed bone with sings of infection (Stage 2) caused by tooth extractions under bisphosphonate therapy (**b**). Despite no large portion of necrotic bone in the maxilla (**c**), Stage 3 is characterized by severe infection leading to maxillary sinusitis in the presented case (**d**).

The clinical presentation of MRONJ was uniformly a white-yellowish areas of exposed necrotic bone, sometimes accompanied erythema of the mucosa or gingival tissue surrounding the lesion (Fig. 2). Uncharacteristic for “classical” Actinomycosis, sinus formation with affection of oro-pharyngeal soft tissue was not noticed for the present patients.

Histological evaluation of the bone specimens collected in the course of surgical treatment showed the presence of *Actinomyces spp.* in 99 of 111 (89%) MRONJ cases. The pathognomonic features of *Actinomyces spp.* infection was found in all histopathological samples, including aggregates composed of *Actinomyces spp.* filaments (sulfur granules), sun-ray morphology of these granules and accompanying inflammatory tissue reaction. Microbiological evaluation of the bone specimens collected in parallel was uniformly negative for *Actinomyces spp.* with only seven exceptions (Table 2). In one MRONJ case with an *Actinomyces*-negative histology result, the pathogen could be cultured from the bone specimen. A diverse microbial flora could be cultured from all but 7 (6%) of the 111 specimens including apathogenic residential oral flora, anaerobic mixed flora and facultative pathogenic microbes. The most commonly facultative pathogens cultured belonged to the families of *Enterobacteriaceae, Neisseriaceae,* and *Streptococcaceae.* The relative frequency of microbes cultured did not differ significantly between *Actinomyces spp.* positive and negative cases of MRONJ.

To evaluate potential risk factors for *Actinomyces*-associated MRONJ, patients were compared with respect to selected demographic and clinicopathological characteristics (Table 3). In this analysis, none of the parameters evaluated separated patients positive for *Actinomyces spp.* clearly from those without detectable infection.

## Discussion

MRONJ affects up to 10% of cancer patients treated with antiresorptive drugs^2^. The disease is associated with significantly increased morbidity and decreased quality of life. Treatment is associated with high rates of treatment failures and recurrences. Prophylactic and therapeutic strategies are impeded by the very limited understanding of pathomechanisms relevant to the evolution of MRONJ^3^,^20^,^21^. In the present study, we substantiate previous evidence that a large majority of MRONJ lesions are infected by *Actinomyces spp*. We failed to identify clear risk factors for *Actinomyces spp.* positivity in MRONJ cases – possibly because of the small number of *Actinomyces spp.* negative cases. As a consequence of these results, current recommendations may have to be revised and adapted to the observation that MRONJ is frequently associated with invasive *Actinomyces spp.* infection.

The detection of *Actinomyces spp.* in 89% of bone specimens by histology is remarkable but may still be a significant underestimation of the factual frequency of MRONJ associated with this infection. Sensitivity of histological evaluation of clinical specimens for microbes is almost universally lower than bacterial culture because the latter involves an amplification step that increases the number of diagnostic targets by several log-titers.

Nevertheless, bacterial culture of bone specimens was negative for *Actinomyces spp.* in all but 6% of samples. *Actinomyces spp.* are fastidious and successful isolation requires culture of specimens under anaerobic or microaerophilic conditions for prolonged periods^22^,^23^. Sensitivity of culture is further reduced significantly by antimicrobial treatment of patients before sample collection^24^,^25^, which was the case in most of the present patients. To increase detection rates by microbial culture, antimicrobial therapy should be discontinued for a few weeks before surgery and collection of clinical samples. Nevertheless, cessation of antimicrobial treatment is difficult to be justified giving the risk of progression and the high sensitivity and specificity of histopathology for the diagnosis of actinomycosis. Antimicrobial treatment before sample collection also changes very likely the oropharyngeal microbiome and microbiological culture results have to be interpreted with caution in bone samples collected from MRONJ cases.

In contrast to microbiological culture, detection of the *Actinomyces spp.* by histology together with pathognomonic sulfur granules and signs of subacute or chronic inflammation, granulation tissue, and osteonecrosis substantiate a possible causal link. *Actinomyces spp.* may be detected reliably in affected bone specimens because of their morphologic appearance upon staining with PAS, Gram and GMS^19^. Nevertheless, histology requires at least several days to provide a reliable diagnosis. In addition, large and high-quality bone specimens are required for a reliable histological diagnosis. The focus of the present study on patients from whom a sufficiently large bone specimen was available for histological evaluation may be associated with a selection bias towards cases of more severe disease that more likely are treated by respective surgery. A rapid diagnosis with epidemiological information on antimicrobial resistance patterns of clinical isolates is important for treatment success. Accordingly, novel assays for the fast and reliable diagnosis of Actinomyces-associated MRONJ are urgently required.

The clinical presentation of “classical”, oropharyngeal actinomycosis and MRONJ-associated actinomycosis differ significantly. Classical actinomycosis manifests clinically as chronic, granulomatous abscess with tissue fibrosis and draining sinuses and unresponsiveness to empiric antimicrobial therapy of a presumed abscess. The face and neck are the most common sites of actinomycosis in humans followed by thoracic, abdomino-pelvic, cerebral and skin infections^26^. Actinomycosis is generally considered an uncommon disease that affects patients of all ages and can appear in both immunocompetent and immunocompromised individuals^27^. In contrast, MRONJ-associated actinomycosis affects primarily cancer patients and is limited in most cases to the jaw bone. Macroscopically visible sulphur granules that are pathognomonic for classical actinomycosis are not visible in MRONJ-associated actinomycosis. Risk factors identified presently in association with detection of *Actinomyces spp.* have to be interpreted with caution in view of the small numbers of *Actinomyces*-negative patients. Hence, MRONJ-associated actinomycosis differs in terms of optimum diagnostic approach and clinical presentation clearly from classical oro-pharyngeal actinomycosis.

To attain an optimum treatment success, the antimicrobial regimen for actinomycosis-associated MRONJ prior to surgery still has to be defined despite the exquisite sensitivity of *Actinomyces spp.* to betalactam agents. Smith *et al*. proposed as possible scheme for the antibiotic treatment of cervicofacial actinomycoses may consist of amoxicillin plus clavulanic acid or also ampicillin plus sulbactam based on the frequent detection of mixed infections^28^. The rational mentioned for this broad-spectrum antimicrobial treatment was poor treatment success achieved in early studies with low-dose penicillin G and a mixed flora of anaerobic and aerobic pathogens commonly recovered along with *Actinomyces spp*^28^. In our experience, however, patients with orocervicofacial actinomycosis respond well to antimicrobial therapy with a betalactam agent only indicative of the low relevance of concomitantly isolated facultative pathogens^23^. Moreover, our present observations may also indicate that cancer patients receiving antiresorptive drugs may benefit from antimicrobial prophylaxis concomitant with dentoalveolar surgery.

Betalactam agents have a high therapeutic index which allows safe administration of high doses of drugs and high therapeutic drug levels are achieved in serum, tissues, bile, and synovial fluid. We found recently that *Actinomyces spp.* isolates are universally susceptible to betalactam antimicrobial agents^15^. Treatment without ß-lactamase inhibitor reduces also significantly the rate of gastrointestinal adverse events such as abdominal discomfort or diarrhea for any cause, including *Clostridium difficile* enterocolitis. Accordingly, we recommend for the treatment of actinomycosis in concordance with others the use of a betalactam antimicrobial agents at high daily doses prior to final surgical treatment of MRONJ^23^. For patients with penicillin allergy, tetracyclines are good alternative for oral therapy, especially in milder disease presentations. In severe infections, carbapenems or the newer compound tigecyclin may be appropriate therapeutic options^15^,^29^.

In conclusion, we found that MRONJ is very frequently associated with the detection of *Actinomyces spp.* by histological evaluation of specimens from necrotic bone. The generally limited sensitivity of histology for the detection of microbes may have further underestimated the factual incidence of Actinomycosis in these patients. The observation of a frequent infection of necrotic bone by *Actinomyces spp.* may suggest that standard surgical treatment of MRONJ may be complemented by antimicrobial treatment.

## Additional Information

**How to cite this article**: Russmueller, G. *et al*. The association of medication-related osteonecrosis of the jaw with *Actinomyces spp*. infection. *Sci. Rep.* **6**, 31604; doi: 10.1038/srep31604 (2016).

## Figures and Tables

**Table 1:** Demographics of the MRONJ study population.

Characteristic	Patients with MRONJ
	(n = 111)
Mean age (SD), years	69 (10)
Gender, no. (%)	
Male	42 (38)
Female	69 (62)
Underlying disease, no. (%)	
Osteoporosis	26 (23)
Cancer	85 (77)
Malignant bone disease, no. (%)	74 (87)
MRONJ Staging, no. (%)	
Stage 0	0 (0)
Stage 1	43 (39)
Stage 2	55 (49)
Stage 3	13 (12)
Location of MRONJ, no. (%)	
Mandible	72 (64)
Maxilla	34 (31)
Both	5 (5)

**Table 2:** Microbiological culture results of bone specimens.

Taxonomic family	***Actinomyces spp.*** detection by histology (n = 111)				OR	p-value*	Adjusted p
	Positive, no. (%)		Negative, no. (%)				
Anaerobic mixed flora	22	(20)	5	(5)	0.4	0.160	1.0
Residential oral flora	25	(23)	5	(5)	0.5	0.301	1.0
*Actinomycetaceae*	6	(5)	1	(1)	0.7	0.561	1.0
*Bacteroidaceae*	8	(7)	1	(1)	1	1.000	1.0
*Clostridiaceae*	4	(4)	0	(0)	Inf	1.000	1.0
*Enterobacteriaceae*	26	(23)	6	(5)	0.4	0.101	1.0
*Enterococcaceae*	5	(5)	0	(0)	Inf	1.000	1.0
*Lactobacillaceae*	7	(6)	1	(1)	0.8	1.000	1.0
*Neisseriaceae*	22	(20)	2	(2)	1.4	1.000	1.0
*Pasteurellaceae*	9	(8)	3	(3)	0.3	0.120	1.0
*Prevotellaceae*	6	(5)	0	(0)	Inf	1.000	1.0
*Saccharomycetaceae*	18	(16)	2	(2)	1.1	1.000	1.0
*Staphylococcaceae*	4	(4)	1	(1)	0.5	0.442	1.0
*Streptococcaceae*	34	(31)	3	(3)	1.6	0.747	1.0
Others	5	(5)	0	(0)	Inf	1.000	1.0
No growth	7	(6)	0	(0)	Inf	1.000	1.0

*Fisher’s Exact.

**Table 3:** Risk factors for detection of *Actinomyces spp.* in MRONJ lesions.

Characteristic	*Actinomyces spp.* detection by histology				OR (95%-CI)	p-value*	Adjusted p
	Positive, no. (%)		Negative, no. (%)				
Bisphosphonate					—	0.830^#^	1.0
Zoledronate	58	(52)	9	(8)			
Combination therapy	19	(17)	2	(2)			
Alendronate	11	(10)	1	(1)			
Ibandronate	6	(5)	0	(0)			
Risedronate	2	(2)	0	(0)			
N/A	2	(2)	0	(0)			
Pamidronate	1	(1)	0	(0)			
Administration route					1.86 (0.22,15.52)	0.540	1.0
intravenous	83	(75)	11	(10)			
oral	14	(12)	1	(1)			
N/A	2	(2)	0	(0)			
Primary disease					0.63 (0.13,3.05)	0.545	1.0
Osteoporosis	24	(21)	2	(2)			
Cancer+	75	(68)	10	(9)			
Type of malignancy							
Breast	24	(21)	5	(4)			
Multiple Myeloma	16	(14)	5	(5)			
Prostate	13	(11)	0	(0)			
Renal	9	(8)	0	(0)			
Lung	6	(5)	2	(2)			
Others	5	(4)	0	(0)			
Malignant bone disease	63	(57)	11	(10)	0.16 (0.02,1.28)	**0.031**	0.744
Chemotherapy					0.84 (0.09,7.45)	0.876	1.0
Current^§^	7	(6)	1	(1)			
Former^$^/Never	92	(83)	11	(10)			
Smoking					6.29 (0.78,50.7)	**0.031**	0.744
Current	36	(32)	1	(1)			
Former$/Never	63	(57)	11	(10)			
Alcohol					1.78 (0.37,8.67)	0.452	1.0
Yes	26	(23)	2	(2)			
No	73	(66)	10	(9)			
Diabetes					1.69 (0.35,8.24)	0.497	1.0
Yes	25	(22)	2	(2)			
No	74	(67)	10	(9)			
Obesity					22028341.47 (0,Inf)	**0.018**	0.450
BMI > 30	18	(16)	0	(0)			
BMI ≤ 30	63	(57)	12	(11)			
N/A	18	(16)	0	(0)			

N/A, data not available; *logistic regression analysis; ^#^chi squared test; +nine patients with cancer additionally suffered from osteoporosis; ^§^three patients additionally received high dosage corticosteroids; ^$^history of chemotherapy or smoking was defined by patients’ state at diagnosis of MRONJ.
